# Neurophotonics in Kyiv, Ukraine

**DOI:** 10.1117/1.NPh.9.2.020101

**Published:** 2022-06-08

**Authors:** Anna Devor

## Abstract

The editorial shares a collective vision for Kyiv becoming a regional resource for teaching, practicing, and advancing neurophotonics - with a recipe for developing a summer school program as a forward-looking way to start.

I love summer schools. As a student, I never got a chance to attend one, but as a teacher I have contributed to many related to neurophotonics. Summer schools combine all of my favorite things: summer, the excitement of doing and teaching immersive science, the joy of seeing friends and colleagues, and interesting destinations. My favorite so far was an IBRO summer school we had in Valdivia, Chile. It was called “Dynamic imaging in neuroscience.” The Valdivia location was chosen by the IBRO president at that time, Pierre Magistretti, because it offered affordable full boarding and travel for Latin American students. The other and equally important reason was a strong local base thanks to Felipe Barros and his laboratory at the Centro de Estudios Científicos.

So, here is a recipe for an awesome summer school:

1. Choose a location with (a) a strong and committed local base but (b) away from the main roads to drive down the registration fees.
2. Find sponsors among nonprofit organizations and industry.
3. Attract world experts to serve as faculty.
4. Select applicants following the principle of inclusivity while giving priority to regional students for whom this school would provide a unique, affordable opportunity.
5. Get together and enjoy science! (Don’t forget to explore the local town and plan some outings with students and teachers.)

Well, I skipped over a few details, but this is the essence.

The secret ingredient for 1a (i.e., a strong local base) is local afficionados and program builders. On that note, I’d like to introduce to you my new Ukrainian friends Pavel Belan, Nana Voitenko, and Olga Garaschuk who have a collective vision of Kyiv becoming a regional resource for teaching, practicing, and advancing neurophotonics.

**Figure:**
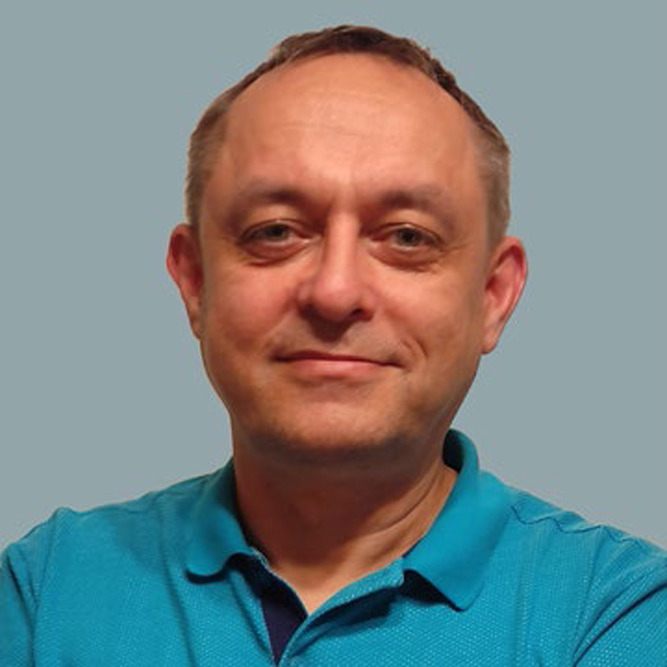
Pavel Belan

Pavel Belan chairs the Department of Biomedicine and Neurosciences of Kyiv Academic University (KAU). Pavel is a physicist by training who specializes in biophysics applied to synaptic transmission and neuronal excitability. His research relies heavily on optical tools such as confocal microscopy and FRET. His vision is to build a thriving, multidisciplinary program in Kyiv that would bring together technology and biology experts under a single umbrella sharing the same neuroscience mission. Such program would attract and retain local talent. After all, there’s no place like home. A strong program combining neuroscience and photonics would be a place to come back to after spending some time abroad.

“At KAU, we believe in transdisciplinary approach,” says Pavel. “We apply physical and chemical approaches to develop new methods and then apply these methods for solving biological problems. Recently, KAU became a partner of the Ukrainian Global University, a new global network that aims to connect Ukrainian students and scholars with educational institutions around the world. We want a strong connection to international community, but we also have to avoid a brain drain. A local Neurophotonics Center in Kyiv serving as a base for an international summer school would achieve multiple goals: push the development of the local base, serve the region, strengthen ties with world experts, raise the standards of local high education. Our ultimate ambition is to lead. Ukraine is a big country. We must leverage the depth of talent available to us at home.”

**Figure:**
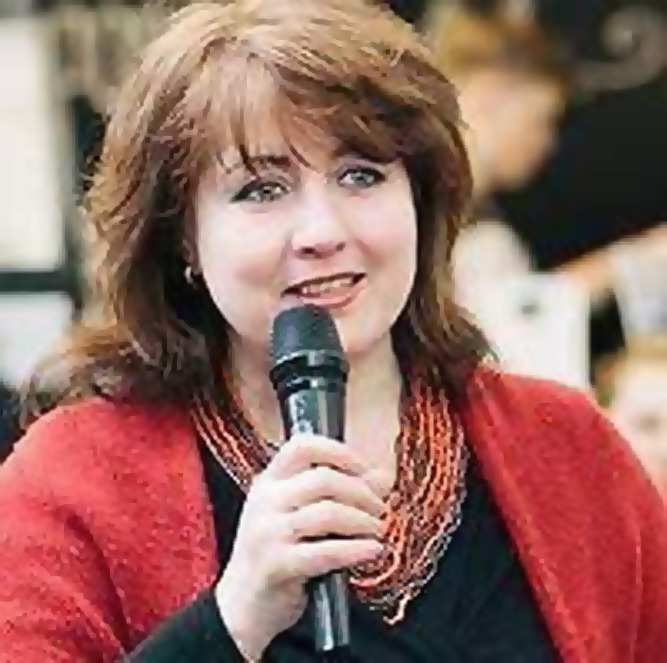
Nana Voitenko

Nana Voitenko is on a mission to establish the Dobrobut Academy Medical School that will be a new kind of medical school in Ukraine. In current practice, Ukrainian medical universities often are disconnected from research centers and hospitals, so students have no exposure to medical research and can receive a diploma without ever seeing a patient. Doesn’t sound like a good idea? Well, because it isn’t. In the West, medical school is followed by years of residency in a real-world clinical setting. Ukraine, however, inherited a Soviet-era system, where researchers and clinicians hardly participate in medical education. Nana is determined to bring a change.

Nana’s own research revolves around calcium imaging in sensory and spinal cord neurons for understanding the mechanisms of chronic pain. She imagines a future where a local neurophotonics resource will serve her medical students. “I see Dobrobut Academy Medical School adopting the Western model of medical education to generate a cadre of world-class medical experts in Ukraine,” says Nana. “Like in the US, some of them will be MD/PhD students who eventually will lead their own laboratories combining medical practice with research. With growing importance of optical tools in biomedical research, related to brain and beyond, building a body of local neurophotonics expertise will play an important role in building and sustaining medical excellence.”

**Figure:**
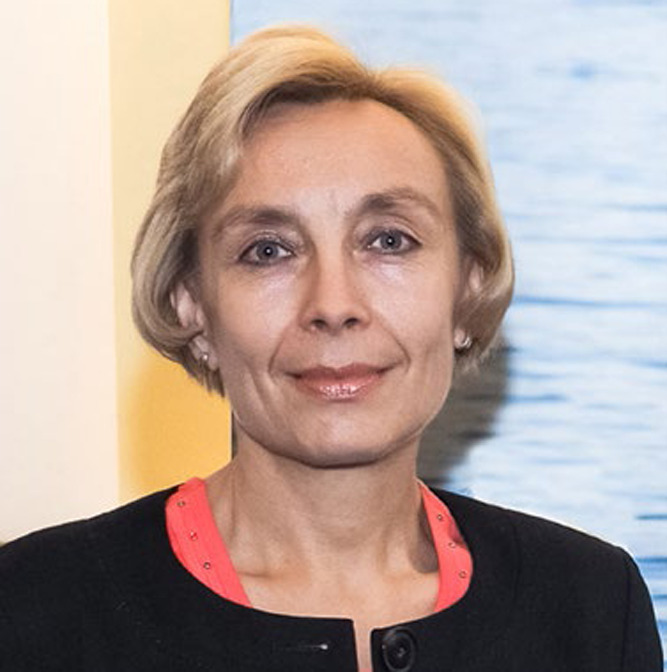
Olga Garaschuk

Olga Garaschuk is a Ukrainian-born neuroscientist living and working in Germany. A professor at University of Tübingen, Olga is widely recognized for her work on two photon calcium imaging in living, behaving brains. In 2003, she published a seminal paper in PNAS that opened the door for delivery of fluorescent calcium probes to many neurons in deep brain tissue at once; today we call it “bulk loading” of synthetic probes. This technique has enabled, for the first time, to image activity of neuronal networks not in a dish but in the brain where they belong! Remember, this was before the advent of genetically encoded fluorescent probes.

Olga is a sought-after teacher and frequent participant of summer schools and short courses in neuroimaging. She has organized and taught several EMBO training courses for live two-photon imaging. As a co-initiator of the Ukrainian Academic International Network (UKRAINET) and the President of German–Ukrainian Academic Society, she organized and participated in several summer schools in Ukraine, the last taking place in Lviv in August 2019. Olga and Nana were busy planning the next one when the COVID pandemic arrived, putting their plans on hold.

“But wait,”—you may say—“forget about COVID, there is a war in Ukraine. Why are we talking about neurophotonics programs and summer schools in the war zone?” Yes, there is a war. Something that was unthinkable just a few months ago has become a brutal reality. But Ukrainian people are resilient. They look to the future, and so should we. Besides, building programs takes time, so we’d better get started!

Here are a few additional useful links:

*ScienceForUkraine* is an academic community group of volunteers who collect and disseminate information about support for Ukrainian students and scholars.

*UAScience.reload* is an initiative of the Ukrainian diaspora who advocate for “preserving, updating and reloading” the Ukrainian scientific community.

